# Assessing daydreaming frequency and control with the Polish version of the Daydreaming Frequency Scale – validation using ecological momentary assessment

**DOI:** 10.3389/fpsyt.2025.1694756

**Published:** 2026-02-04

**Authors:** Michał S. Skorupski, Izabela Krejtz, Monika Kornacka

**Affiliations:** 1 Emotion Cognition Lab, SWPS University, Katowice, Poland; 2 Institute of Psychology, SWPS University, Warsaw, Poland

**Keywords:** daydreaming, Daydreaming Frequency Scale, validation, psychometric values, ecological momentary assessment, task-unrelated thoughts

## Abstract

Daydreaming is a prevalent type of cognitive activity, in which the content of one’s thoughts is unrelated to the current task. The Daydreaming Frequency Scale (DDFS) was the first questionnaire devised to measure the occurrence of daydreaming and remains widely used for this purpose today. We report on three studies validating the Polish version of the DDFS. Study 1 (n=385) examines its factor validity, internal consistency, and criterion validity (tested by computing correlations with the scores of other questionnaires measuring similar phenomena and known consequences of daydreaming). Study 2 (n=1301) confirms the factor structure established in Study 1 and provides additional test–retest analyses conducted over a three-month interval. Study 3 (n=214) tests the link between the trait-level DDFS score and daily occurrence of task-unrelated thoughts (TUTs) and the degree of everyday control over such thoughts, using ecological momentary assessment (EMA). During the factor validity analysis, we tested the possibility of implementing a two-factor structure solution; however, after careful consideration, the original one-factor structure was retained. The Polish DDFS demonstrated high internal consistency (Cronbach’s α = .92). The results of the correlation-based criterion validity testing were also satisfactory. The findings of Study 3 suggest that DDFS score is positively related to actual daily TUTs occurrence and negatively related to daily thought control, further supporting the good criterion validity of the questionnaire.

## 1 Introduction

Everyone knows the feeling of trying to focus on a task, only to realize a moment later that for the past few minutes they have been immersed in thoughts fully unrelated to their current activity and surroundings. This process is known as daydreaming and as it is very common, happening to every person and being estimated to make up 30 to 46.9% of our daily thought content (1, 2). It can influence our lives strongly, both in a constructive and unconstructive manner.

As with most complex cognitive phenomena, daydreaming has its functions, but it can also come at a cost (3). It can help us facilitate creative processes (4), plan for the future (5), and strengthen the ability to delay gratification (6). It also seems to play a positive part in socio-emotional development (7) and more general social cognition (8). On the other hand, some studies indicate that daydreaming can be linked to a decline in general emotional state (2), a rise in depressive and anxiety symptoms (9), and other psychopathological phenomena, such as obsessive-compulsive symptoms and dissociation (10). It is a significant risk factor in work and driving related accidents (11), and it can hinder the positive effect of waking rest on memory consolidation (12). Maladaptive daydreaming often coexists with attention deficit hyperactivity disorder, anxiety, depression, and obsessive-compulsive disorder (13). Some studies suggest that daydreaming might sometimes be considered as a form of behavioral addiction (14, 15).

To discuss the reasons for such a differentiated outcome of this cognitive phenomenon, it is important to define it. Jerome L. Singer, co-author of the Daydreaming Frequency Scale (16), who is referred to by some researchers as the ‘father of daydreaming’ (17), himself regarded daydreaming as a phenomenon very difficult to precisely define (18). He did, however, provide a concise description of it (18, p.3)

*“[ … ] daydreaming represents a shift of attention away from some primary physical or mental task we have set for ourselves, or away from directly looking at or listening to something in the external environment, toward an unfolding sequence of private responses made to some internal stimulus.”*

Nevertheless, a ubiquitous and uncontroversial definition of daydreaming remains elusive, especially with so many terms used to describe phenomena either equivalent or similar to it (19), such as task-unrelated thoughts (TUTs) (20), mind-wandering (1), spontaneous thoughts (21). Some of the differences in the understanding and differentiation of these terms are subtle, but in other cases the views of some authors seem almost contradictory – i.e. Giambra (20) stated that daydreaming is always unintentional, contrary to mind-wandering, which can be deliberate, while Dorsch (22) sees daydreaming as an effect of voluntary mental agency, in contrast to mind-wandering, which is seen as far less controlled.

It is important to mention that the scales constructed by Singer and Antrobus (16, 23), devised to measure daydreaming, accommodate a very wide range of spontaneous thoughts – from acceptable to unacceptable, vivid to hallucinatory, visual and auditory, past/present/future oriented, task-distracting and not, internally and externally focused, problem-solving, hostile, heroic, aggressive, with different levels of absorption or repetitiveness, pleasantness, unpleasantness, and many other types. Mind-wandering is even explicitly treated as a subtype of daydreaming in the Imaginal Processes Inventory (16). Together with the above-mentioned description, this suggests that the authors of DDFS understood daydreaming as an overarching umbrella term, similar to what Marchetti et al. (21) understand as spontaneous thoughts or what Giambra (24) sees as TUTs. This approach also seems compatible with the family-resemblance approach, proposed by Seli et al. (25), in which all of the above-mentioned phenomena are treated as one heterogeneous, gradable concept, with different characteristics. As this article is mostly focused around the DDFS, we use the term daydreaming together with its *designata* in accordance with the way Singer and Antrobus (16, 26) proposed at the moment of the scale’s original publication – as thoughts that are task unrelated, without differentiating them on the basis of intentionality, stimulus-dependence or emotional valence.

The question of what makes daydreaming helpful and adaptive for some people in some situations and what makes it harmful for others in other situations, is a subject of debate and a topic of many research articles. There are two main hypotheses as to what indicates adaptivity or non-adaptivity of daydreaming. The first – the content regulation hypothesis (27) – suggests that the differentiating factor is the thought content itself. According to the second – the context regulation hypothesis (28) – daydreaming is adaptive when happening during a cognitively undemanding task, but maladaptive, and an effect of cognitive control failure in situations requiring a high level of cognitive activity. Carciofo and Jiang (29) noted more positive effects of deliberate than spontaneous daydreaming. Low sleep quality (30) and prolonged stress (31) may be linked to more negatively valenced and maladaptive daydreaming.

Recently, research on daydreaming and related phenomena seems to have attracted a new wave of attention from the scientific community (32, 33). In line with this trend, the Daydreaming Frequency Scale, which was first used as a separate questionnaire by Giambra almost 30 years ago (24), but originates from the even older Imaginal Process Inventory (16), has been adopted, validated, and used in French (34), Dutch (9), Japanese (35), and German (36) versions. Lately, two other questionnaires, measuring different but related phenomena had their Polish versions published: Maladaptive Daydreaming Scale (37) and Mind Wandering Questionnaire (38).

Herein we report on the validation of the Polish version of the Daydreaming Frequency Scale in three studies. In the first study, we analyze the psychometric properties of the questionnaire, using Factor Analysis, internal consistency measures, and criterion validity analysis. The second confirms the factor structure established in Study 1 and provides additional test–retest analyses conducted over a three-month interval. The third study aims to measure the extent to which the trait-level score of DDFS is linked to the actual frequency of occurrence of TUTs and the degree of control exerted over such thoughts in everyday situations. This is the main contribution of this article, as other validations did not test the validity of DDFS in the daily context. For this purpose, we use an experience sampling method, which is especially valuable when examining the content and context of everyday thoughts and the dynamics between them, as it is more ecologically valid and rich in contextual information than laboratory measurements, and at the same time more reliable than retrospective measures (1, 39).

With the growing number of studies concerning daydreaming and related phenomena, and with the ongoing debate on the definitions and interrelations between the processes mentioned above (e.g., daydreaming, TUTs, mind-wandering, spontaneous thoughts), we believe that providing access to a tool enabling the direct measurement of daydreaming frequency in the Polish population is of particular importance, as it will facilitate further research on these phenomena within the Polish context and allow for cross-cultural comparisons. Additionally, the use of EMA in the validation process should widen the understanding of the relation between the declared questionnaire-measured daydreaming frequency and the actual intensity of daydreaming in participants’ everyday lives.

## 2 Study 1

In the first study, the psychometric properties of the Polish version of DDFS were examined by the following steps. First, the factor structure of the scale was explored using Exploratory Factor Analysis. The resulting factor solutions were then evaluated with Confirmatory Factor Analysis. Next, the internal consistency of the scale was assessed using Cronbach’s α. Finally, to test criterion validity, correlational analyses were conducted between the DDFS and questionnaires measuring different forms of TUTs, as well as symptoms of anxiety and depression.

### 2.1 Materials and methods

#### 2.1.1 Participants

450 adult participants from a non-clinical population took part in the study. The final sample consisted of 385 participants (*Mean age* = 30.36, *SD* = 17.88, 291 females, 90 males and 4 of other genders) who correctly filled out all the questionnaires and did not withdraw from the study. The sample size is sufficient according to Comrey and Lee (40) standards. The relatively high dropout rate may have been caused by the high number of questionnaires used in the study. The participants were recruited between October 2020 and March 2021.

#### 2.1.2 Procedure

Participants were recruited through social media. After giving an informed consent, they completed the questionnaires listed in the Measures section. The procedure was conducted online, using Qualtrics software. The participants who were students of SWPS University at the time of the study, received credit points for their participation. All studies described in the article were reviewed and approved by Research Ethics Commission of SWPS University (IRB protocol number WKEB69/03/2021).

#### 2.1.3 Measures

*Daydreaming Frequency Scale (DDFS).* The DDFS is a 12-item scale, measuring the frequency of daydreaming (23, 24). Each of the items enquires either directly about the frequency of daydreaming (e.g., ‘I recall or think over my daydreams…’, with anchors ranging from ‘A. infrequently.’ to ‘E. many different times during the day.’) or about the proportion of time spent on daydreaming versus other activities in specific situations (e.g. ‘Instead of noticing people and events in the world around me, I will spend approximately …’ with anchors ranging from ‘A. 0% of my time lost in thought.’ to ‘E. 50% of my time lost in thought.’). Participants choose the answer that best fits them on a 5-point scale, with anchors differentiated for each item based on its specific content. The DDFS score is obtained by adding the points from all the items. The scale was first developed as a part of the Imaginal Process Inventory (16) and later adapted for use as a separate questionnaire by Giambra (24).

The DDFS scale was translated, using a back-translation protocol. The questionnaire was translated by a bilingual psychology expert and back-translated by another bilingual expert. The two English versions were assessed to be similar, independently by two judges – one holding a PhD degree, the other a Master’s degree in Psychology. The translated questionnaire is presented in the **Supplementary Materials**. The psychometric properties are reported in the Results section.

*Perseverative Thinking Questionnaire (PTQ).* The PTQ is a 15-item scale assessing the level of perseverative thinking (41). It has one higher-order factor (a total score capturing repetitive negative thinking; RNT) and three lower-order factors: core characteristics of RNT (e.g., ‘The same thoughts keep going through my mind again and again.’), unproductiveness of RNT (example item: ‘I think about many problems without solving any of them’), and RNT capturing mental capacity (example item: ‘I can’t do anything else while thinking about my problems’). The participants indicate to what extent they agree with the statements on a Likert-type scale ranging from 1 (‘never’) to 5 (‘almost always’). The Polish version of PTQ (42) was used. In our study, Cronbach’s α was .96 for the higher-order factor, .95 for the core characteristics factor, .82 for the unproductiveness of RNT factor, and .84 for the capturing mental capacity factor.

*Rumination Response Scale (RRS).* The RRS is a 22-item scale assessing depressive rumination (43). It consists of a higher-order factor, which is global depressive rumination score, and two lower-order factors: brooding (example item: ‘How often do you think about how alone you feel?’) and reflection (example item: ‘How often do you analyze recent events to try to understand why you are depressed?’). The participants indicate how often they use the emotion regulation strategies described in the items on a 4-point Likert-type scale ranging from 1 (‘almost never’) to 4 (‘almost always’). The Polish version of the RRS (42) was used. In our study, Cronbach’s α was .93 for the global depressive rumination score, .78 for the brooding factor, and .75 for the reflection factor.

*Hospital Anxiety and Depression Scale (HADS).* The HADS is a 14-item scale, with seven items measuring depression (e.g., ‘I have lost interest in my appearance’ with anchors ranging from 1- ‘definitely’ to 4 ‘I take just as much care as ever’) and seven items measuring anxiety (e.g., ‘I get sudden feelings of panic’ with anchors ranging from 1 ‘very often indeed’ to 4 ‘not at all’) (44). The participants indicate how frequently they feel or act in a way described in the item by choosing an answer on a 4-point Likert-type scale, with anchors differentiated for each item based on its specific content. The Polish version of the HADS (45) was used in our study. In our study, Cronbach’s α was .75 for the depression scale and .83 for the anxiety scale.

*Future Self-Thoughts (FST) questionnaire.* The FST (46) is an 11-item scale, with 6 items measuring frequency (e.g. ‘I often picture myself in the future in different ways and think about the various paths that could lead me to those different futures’) and 5 items measuring clarity (e.g. ‘My future seems vague and uncertain to me’) of participants’ thoughts about themselves in the future. Participants answer to what extent they agree with an item on a Likert-type scale, ranging from 1 (‘not at all true for me’) to 6 (‘completely true for me’). In our study, Cronbach’s α was .78 and .86, respectively.

Because of technical issues, one of the items of the Future Self-Thought questionnaire was not collected from the participants, which may have influenced the score on the ‘frequency’ scale. As a result, the ‘frequency’ and ‘clarity’ scales used in our study had an equal number of 5 items. As stated above, the internal consistency level remained consistent with the original and full version of the questionnaire (46).

#### 2.1.4 Analytical plan

In order to explore the possible factor structure of the Polish version of DDFS, exploratory factor analysis (EFA) was conducted with the use of ‘Psych’ (47) and ‘GPArotation’ (48) packages in R (30). For the Exploratory Factor Analysis, eigenvalues had been extracted for the data based on Principal Axis Factor (PAF) analysis. There are 3 most commonly used methods of using eigenvalues to determine the number of factors (49). According to the first, known as the Kaiser-Guttman criterion, the factors whose eigenvalues are higher than 1 should be retained (50, 51). In the second popular method, known as the scree test (52), eigenvalues for each factor are plotted into a scree plot, with eigenvalue level on the X axis and factor number on the Y axis. A visual evaluation is carried out to determine at which point both the value and its variability have visibly fallen. Both of these methods, although widely used, are considered inaccurate by today’s standards (53). The third way to analyze eigenvalues for factor retention is parallel analysis (PA) (54, 55). In this method, random data sets are generated, then eigenvalues from both random and real data are extracted, averaged, and compared. Factors whose eigenvalues are higher than the eigenvalues of their respective counterparts extracted from random data are retained. Parallel analysis is considered one of the most accurate ways to use eigenvalues for factor retention (53, 56).

The factor loadings for all considered factor solutions were obtained using PAF with the oblimin rotation using the psych package in R. For an item to be considered satisfactory, it should load onto their primary factor above .40 and load onto other factors below .30, with the difference between primary and alternative factor loading being over .20 (57).

In the next step, Confirmatory Factor Analysis (CFA) was used to check the validity of all factor structure models, that were considered as a result of the EFA. In CFA, the number of factors and the association of the specific items to them is specified *a priori*; the fit of this model is then tested (58). The most commonly reported fit indexes are the comparative fit index (CFI), the root-mean-square error of approximation (RMSEA), and the standardized root-mean-square residual (SRMR) (59). The values considered as an acceptable fit are >.95 for CFI, <.08 for RMSEA and ≤.1 for SRMR (60). However, some sources suggest a CFI between .90 and .95 should be considered acceptable, and CFI over .95 considered good (61, 62). CFA was performed using the Lavaan package (63) in R.

The internal consistency was evaluated by computing Cronbach’s α. Item reliability statistics were computed for all items assigned to each factor (the results are provided in the Supplementary Materials in **Supplementary Tables S1**-**S4**). Finally, the criterion validity was evaluated with Pearson’s correlation coefficient between the DDFS and the corresponding questionnaires listed above. We expect that the DDFS score should be positively correlated with the scores of the already validated measures of other TUTs types (PTQ, RRS, FST frequency). As in the past studies, intensity and frequency of TUTs were associated with lower mood, higher anxiety and depression levels (2, 9, 13), we also expect the DDFS score to correlate positively with HADS Anxiety and Depression scales. A negative correlation is expected between the DDFS score and FST clarity, as suggested in the validation of the French version (34).

### 2.2 Results

#### 2.2.1 Exploratory factor analysis

**Figure 1:**
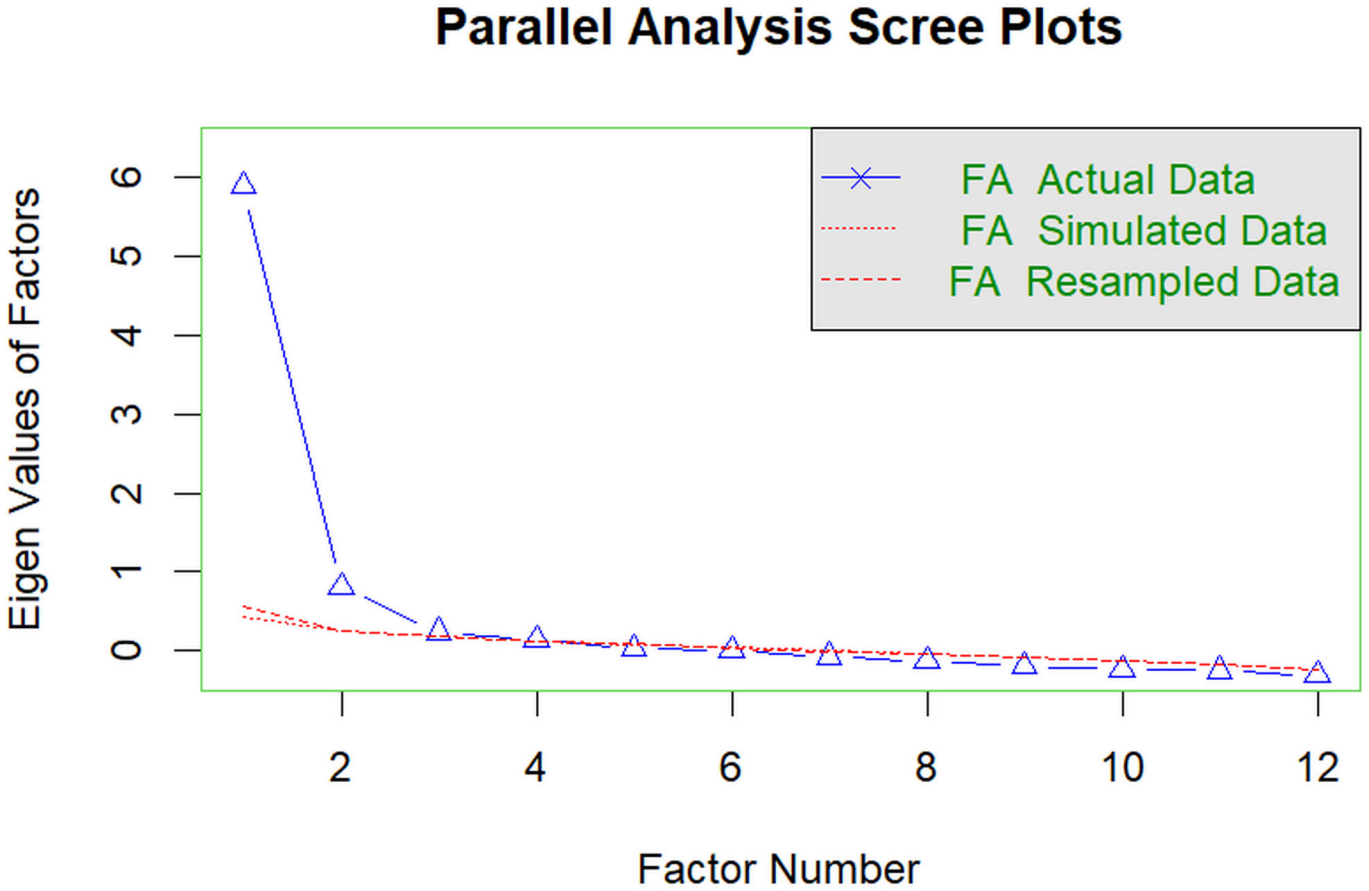
Scree plot of the parallel analysis results.

The results of the parallel analysis indicated a three-factor solution as the most suitable (see **Figure 1**), however the pronounced drop from the first to the second eigenvalue may suggest a strong general factor underlying the scale (64). For this reason, and because the original version of the scale is based around a single-factor solution, both the three-factor and single-factor solutions, along with a hybrid model were explored in the next analyses.

The factor loadings obtained for both three-factor and one-factor solutions are presented in **Table 1**. The one-factor solution explained 49% of the variance, while the three-factor solution explained 63%. All loadings in the one-factor solution exceeded .60. In the three-factor solution, the loadings for items 11 and 12 were not satisfactory (57). However, a removal of these items would strongly disrupt the structure of the questionnaire, making it difficult to compare the results of the Polish version of DDFS to the original scale, as well as previously published adaptations. Therefore, both items were retained for further analyses.The three-factor model was interpreted based on the content of the items loading into each of the factors, in search of a theoretical explanation of the new differentiation.

**Table 1:** Factor loadings for the one-factor and the three-factor solutions.

Item	One-factor model	Three-factor model		
	1	1	2	3
DDFS_1	.77	.92	–	–
DDFS_2	.84	.46	.38	–
DDFS_3	.62	–	.69	–
DDFS_4	.79	.81	–	–
DDFS_5	.72	.32	.51	–
DDFS_6	.55	–	.83	–
DDFS_7	.79	.53	.39	–
DDFS_8	.63	–	.73	–
DDFS_9	.66	–	.-	.50
DDFS_10	.65	–	–	.91
DDFS_11	.68	–	.32	–
DDFS_12	.62	–	–	.33

Loadings lower than .30 were omitted in the table

The items with highest loadings on Factor 1 (DDFS 1, DDFS 2, DDFS 4, DDFS 7) consist of short and direct questions regarding the frequency of daydreaming (*e.g.* ‘Daydreams and fantasies make up [ … ] % of my daily thought content’). Their content does not carry information on whether the act of daydreaming described is intentional (as in items 9 and 10). It also does not characterize daydreaming as a consequence of inattentiveness to ongoing tasks or the surrounding environment (as in items 3, 5, 6, 11).

The items grouped under Factor 2 (DDFS 3, DDFS 5, DDFS 6, DDFS 8, DDFS 11) are focused around daydreaming that occurs during – or instead – of a currently undertaken activity (*e*.*g*. ‘When I’m not paying attention to a job, book, or TV I tend to be daydreaming…’). The act is described as ‘drifting away’ (Items 3 and 5; this wording appears in the Polish version of the DDFS, as discussed later in Section 2.3.1) or as occurring in place of (a) noticing elements of ones’ surroundings (item 6), or (b) paying attention to an ongoing activity (item 11). These items describe daydreaming as an effect of executive control failure (28). Item 8 does not fully fit this criterion – it directly enquires about the proportion of participants’ daytime occupied by recalling the past, imagining the future or thoughts of abstract nature. Perhaps the strong loading of item 8 on Factor 2 may be explained by participants’ viewing the described thought content as useless or unwanted, and therefore treating it as another instance of executive control failure.

The items loading on Factor 3 (DDFS 9, DDFS 10, DDFS 12) enquire about active and intentional daydreaming (items 9 and 10) or daydreaming in the context of a long journey by means of public transport (item 12) – a situation associated a low cognitive load. This suggests, that Factor 3 measures daydreaming that is not task-disruptive, and should therefore have vastly different effects to Factor 2.

The loadings of items 2, 5, and 7 did not fully meet the criteria of the difference between primary and secondary loadings. However, as item 5 enquires directly about engaging in daydreaming at a cost of being focused on another task, while items 2 and 7 do not reflect such a trade-off, we decided to include item 5 only in Factor 2, and items 2 and 7 only in Factor 1. This solution ensures that each item was assigned to the factor it conceptually best represents, while also allowing us to retain a simple and more interpretable factor structure. Similarly, we decided not to exclude item 11 from Factor 2 – as, although the loading of item 11 on Factor 2 is low (.32), its content (daydreaming instead of focusing on a meeting or a show) fits well within the proposed theoretical conceptualization of Factor 2.

Based on the interpretation of the item content loading on each factor, a provisional categorization was established: Factor 1 – Frequency of Daydreaming**;** Factor 2 – Task-Disruptive Daydreaming**;** and Factor 3 – Non-Task-Disruptive Daydreaming.

#### 2.2.2 Confirmatory factor analysis

Based on the results of the parallel analysis and the content-based interpretation of items loading on each of the three potential factors in the three-factor solution, three models were evaluated using CFA. First, a single-factor model was examined, as this structure was applied in the original DDFS. Second, a three-factor model, suggested by the EFA results, was tested. Finally, a bi-factor (general-specific) model was included, incorporating both a General Score Factor (with all items loading on it) and the three Specific Factors described above. In this solution, no hierarchical relation between the General Score Factor and the Specific Factors exists (64). The CFA results for all three tested solutions are presented in **Table 2**.

**Table 2:** Results of CFA for the single-factor, three-factor and bi-factor models.

Model	CFI	RMSEA	SRMR
Single-factor model	.819	.155	.072
Three-factor model	.886	.126	.076
Bi-factor model	.963	.082	.034

For the bi-factor model, orthogonal constraints between the general and specific factors were imposed, to ensure statistical independence and to isolate group-specific residual variance. Results without orthogonal constraints are reported for readers’ information (CFI = 0.97; RMSEA = 0.077; SRMR = 0.03).

Only the general-specific bi-factor model met the established criteria for CFI and SRMR, and was close to meeting the criterion for RMSEA. This model is in line with the parallel analysis results, which suggested an existence of one stronger factor, possibly underlying the others. Finally, this solution allows us to further explore the newly found differentiation, while still being able to provide a General Score, consistent with other versions of DDFS.

#### 2.2.3 Internal consistency and item reliability

The Polish version of the DDFS demonstrated high internal consistency, with Cronbach’s α = .92 and with no possibility of raising the score by removing any of the items. This result is also very close to the Cronbach’s α for the original DDFS, of .91 (24). For each of the three Specific Factors included in the analysis the Cronbach’s α was: .9 in Specific Factor 1, .83 in Specific Factor 2, .77 in Specific Factor 3. The results of item reliability tests (available in Supplementary Materials in **Supplementary Tables S1**-**S4**) suggest that removing any of the items from the proposed factors would not increase the homogeneity of the scale.

#### 2.2.4 Criterion validity

As shown in **Table 3**, the General Score of the Polish version of DDFS significantly correlates with other instruments measuring TUTs; positively with the Perseverative Thinking Questionnaire and Ruminative Response Scale, Future Self Thought Frequency scale and negatively with the Future Self Thought clarity scale. The DDFS score showed a low, but statistically significant positive correlation with the depression and anxiety scale of the HADS.

**Table 3:** Correlations between DDFS, other spontaneous thought, depression and anxiety measures.

Measure	1	2	3	4	5	6	7	8	9	10	11	12	13	14
1. DDFS General Score	1													
2. DDFS_F1	.93	1												
3. DDFS_F2	.88	.7	1											
4. DDFS_F3	.83	.72	.57	1										
5. PTQ General Score	.52	.39	.65	.29	1									
6. PTQ F1	.5	.37	.62	.26	.97	1								
7. PTQ F2	.49	.37	.59	.29	.9	.82	1							
8. PTQ F3	.46	.32	.59	.25	.86	.75	.75	1						
9. RRS Reflection	.37	.28	.41	.27	.41	.4	.33	.37	1					
10. RRS Brooding	.43	.33	.47	.34	.63	.6	.6	.53	.5	1				
11. HADS Anxiety	.31	.2	.44	.12*	.66	.63	.62	.57	.4	.55	1			
12. HADS Depression	.31	.22	.43	.14*	.63	.61	.59	.56	.39	.54	.83	1		
13. FST Frequency	.47	.38	.46	.38	.42	.42	.39	.33	.3	.39	.33	.3	1	
14. FST Clarity	-.29	-.23	-.38	-.12*	-.51	-.46	-.56	-.45	-.27	-.37	-.48	-.47	-.21	1

*p<0.05. All correlations without an asterisk had p<0.001.

Although the three specific factors correlated with the other scales in the same direction as the General Score Factor, the correlations involving Specific Factor 1 and Specific Factor 3 were consistently weaker, whereas those involving Specific Factor 2 were consistently stronger than the correlations observed for the General Score Factor. The correlations between Specific Factor 3 and HADS Anxiety (*r* = .12; *p* = 0.039), HADS depression (*r* = .14, *p* = 0.021), as well as FST Clarity (*r* = .12; *p* = 0.039) are very weak.

### 2.3 Discussion

The results of Study 1 provide a rationale for adopting a different factor structure to the Polish version of the DDFS, in comparison to the original scale. Based on EFA, a general -specific bi-factor model was constructed that enabled the retainment of the General Score Factor (useful for comparing results with the original scale and other published adaptations), while introducing three Specific Factors (Frequency, Task-Disruptive Daydreaming, Non-Task-Disruptive Daydreaming), allowing for a more fine-grained interpretation of the results. The results of CFA show that the general-specific bi-factor model is characterized by a good fit.

The existence of cross-loadings between items assigned to Factor 1 and Factor 2 may suggest that the theoretical constructs underlying these two factors are more closely related to each other than to the construct represented by Factor 3.

The fact that Specific Factor 2 (Task-Disruptive Daydreaming) shows the strongest, while Specific Factor 3 (Non-Task-Disruptive Daydreaming) the weakest correlations with measures of maladaptive TUT types (perseverative cognition and rumination), as well as with depressive and anxiety symptoms, seems to corroborate the theoretical differentiation between the two factors. If the items loading on Specific Factor 2 measure the type of daydreaming that can be seen as an executive control failure, its stronger link to maladaptive TUTs and psychopathological symptoms, would be in line with the Context Regulation Hypothesis (28). Conversely, intentional (65), controllable (66), and freely moving (32) forms of mind-wandering/daydreaming have been shown to be less strongly associated with negative outcomes – characteristics that appear consistent with the thought patterns captured by Specific Factor 3.

#### 2.3.1 Semantic differences and factor structure

The difference in factor structure between the original and the Polish version of DDFS may be partially influenced by the semantic differences in particular items. The Polish language does not contain a direct translation of the English word ‘daydreaming’. The word ‘marzenie’ seems to be the closest equivalent, but it is more often used as a noun, than a verb. This may be the reason why, in our translation procedure, when ‘daydreaming’ was used in a longer sentence and in the context of other verbs, it was more natural for the translator to change the structure of the sentence so that ‘daydream’ could be used as a noun and another verb was used to describe the action of engaging in it.

In items 1, 7, 11 and 12, the translation of the original ‘I daydream’ is ‘Oddaję się marzeniom’ – the meaning of which would be closer to ‘I indulge in…’, ‘I surrender myself to…’ or ‘I give myself to…’ daydreams. This way of constructing the sentence is more natural in the Polish language, but the reception of the sentence may slightly differ – as the subject is now performing the act of ‘indulging in’ or ‘giving oneself to’ the act of daydreaming, rather than just performing the act of daydreaming.

For a similar reason, items 3 and 5 contain a short description of ‘thoughts drifting away’ (‘towards the world of daydreams’ in the case of item 5). The Polish construction of these two items seems to carry a stronger sense of uncontrollability of the thought process (‘I tend to be daydreaming’ versus ‘My thoughts tend to drift away towards the world of daydreams’), as well as a stronger separation from the current task. This difference may have influenced the loadings on Specific Factor 2, as the lower thought control and stronger separation from the task described in items 3 and 5 may have made these items more similar to one another, and more thematically coherent with other items enquiring about daydreaming as an effect of inattentiveness.

## 3 Study 2

The results of Study 1 pointed towards a factor structure that differs from the original DDFS. As conducting a CFA on an independent sample (rather than the one used for the EFA) is necessary to avoid overfitting and provides a stronger test of replicability, in Study 2 we evaluated the proposed structure using data from another, previously published study (67).

### 3.1 Materials and methods

#### 3.1.1 Participants and procedure

1301 participants (1110 female, 169 male, 16 other, 6 participants did not provide information on gender, *M_ag_*_e_=25.72, *SD* = 8.04), recruited through the university research recruitment system, filled out a series of questionnaires in Qualtrics at Time 1. Of these, 397 (353 female, 40 male, 2 other, 2 did not provide information on gender, *M_ag_*_e_=25.72, *SD* = 8.32) filled out the questionnaires three months later (Time 2). The participants received points in the university credit system to compensate their participation.

#### 3.1.2 Measures

The participants filled out the Polish version of DDFS (described in Study 1). The description of other measures (not included in the analyses performed in Study 2), used in the study can be found in the published article (67).

#### 3.1.3 Analytical plan

First, the factor structure established in Study 1 was tested using CFA, following the same analytical procedure. CFA was conducted on the results of the Polish version of DDFS filled out by participants in T1.

Additionally, a test-retest reliability assessment between T1 and T2 was conducted, to explore the temporal stability and consistency of scores across repeated measures. Intraclass Correlation Coefficient (ICC) – a measure of consistency between repeated measurements – was calculated (68). Standard Error of Measurement (SEM) was estimated to evaluate score precision (68). To establish the threshold of actual change beyond measurement error, the Minimal Detectable Change at 95% Confidence Interval (MDC_95_) was computed (69). Finally, a paired-samples t-test comparing DDFS scores at T1 and T2 was conducted to assess potential systematic bias. All of the above mentioned analyses were conducted for both the General Score and the three Specific Factors separately.

### 3.2 Results

The results of the CFA (*N* = 1301; CFI = .966; *RMSEA* = .079; *SRMR* = .028) indicated an acceptable model fit. These findings confirm the factor structure proposed as a result of the analyses conducted in Study 1.

Test–retest reliability of the General Score of DDFS indicated moderately good temporal stability (*ICC* = .71; *95%CI*: 0.66–0.75). The results of SEM (4.61) and MDC95 (12.8), suggest that only relatively large changes (≈13 points, with the minimum score of 12 and maximum score of 60) can be seen as a result of meaningful change, rather than measurement noise. No systematic mean-level change between sessions was uncovered (*t* = 2; *p* = .10), which suggests that the score of the Polish version of DDFS stayed generally stable over the three months between T1 and T2.

The results of test-retest analyses for each of the three Specific Factors are presented in **Table 4**. All of the factors showed moderate temporal stability. A mean-level change between the scores in T1 and T2 was observed only for Specific Factor 2.

**Table 4:** Results of test-retest reliability measures for Specific Factors 1–3.

Factor	*ICC*	*95% CI*	*SEM*	*MDC95*	*t*	*p*
Specific Factor 1	.66	.6-.71	2.01	5.58	1.08	.282
Specific Factor 2	.62	.55-.68	2.07	5.74	2.1	.036
Specific Factor 3	.66	.6-.72	1.4	3.89	0.23	.818

### 3.3 Discussion

The results of Study 2 support the validity of the factor structure established in Study 1. Additional test-retest analyses indicated that the Polish version of DDFS demonstrates moderately good temporal stability, with no significant changes between the scores in T1 and T2 for the General Score, Specific Factor 1 and Specific Factor 3. The statistically significant decrease observed in scores of Specific Factor 2 may indicate that the measure of Task-Disruptive Daydreaming is partially state-dependent.

## 4 Study 3

In Study 3, we analyzed the relationship between the General Score on the DDFS (which measures trait-level daydreaming frequency), scores of each of the newly proposed Specific Factors and the frequency and controllability of daily TUTs, measured by ecological momentary assessment methods (EMA). The goal of this analysis was to further expand the criterion validity testing in a strongly ecologically valid manner.

### 4.1 Materials and methods

#### 4.1.1 Participants

279 adult participants from a non-clinical population took part in the second study. The final sample consisted of 214 participants (Mean age = 29.17, SD = 8.93, 169 females, 43 males and 2 did not specify the gender) who finished the study with a compliance higher or equal to 60%, which was set as a minimum. The participants in Study 3 were recruited between January and December 2021.

#### 4.1.2 Procedure

Participants were recruited through social media. After signing the consent form, they filled in the DDFS and a demographic information survey using the Qualtrics platform. The participants were then contacted by an experimenter, who explained the procedure of the study. After installing the Movisens application (movisens GmbH, Karlsruhe, Germany) on their smartphones, the participants took part in the EMA part of the study, which lasted for 7 days. The application was set to send 7 semi-randomly timed beeps a day to each participant. The beeps appeared in 2-hour time slots, scattered throughout a 14-hour window of daily activity, which was earlier set by the participant. The participants had 20 minutes to answer the EMA questions; after that time expired the beep was counted as missed.

The study design required participants to generate an individual identification code (according to the provided instructions) and to enter it in a consistent format when completing the questionnaires and again at the beginning of the EMA procedure. For 13 participants, the codes did not match across the two study components, which prevented data linkage and consequently contributed to an increased dropout rate.

#### 4.1.3 Instruments

The EMA measures included 2 questions adapted from Kornacka et al. (70). The first measured the occurrence of TUTs in the given moment: ‘Just before the beep, your thoughts were …’, with a visual analogue scale (VAS) ranging from 0 (‘task-related’) to 100 (‘task-unrelated’). The second measured thought control, revealing the level of control the participants have over their thoughts in the given moment: ‘Just before the beep, to what extent were your thoughts possible to control’, with a VAS ranging from 0 to 100.

The Daydreaming Frequency Scale (23), described in the first part of this article, was used as the only instrument measuring trait-level characteristics.

#### 4.1.4 Analytical plan

The data were analyzed using a multilevel analysis with HLM 8.0 software. EMA measures were treated as Level 1 variables and nested in participants (Level 2). The day level was omitted as in previous studies concerning daydreaming, which used multilevel analysis (70). The DDFS score, as a trait-level measure, was assigned to Level 2. Level 1 variables were entered to the model group mean centered; DDFS was grand mean centered. All coefficients were computed with robust standard errors and based on a fixed model.

The mixed models used for the calculation of the relationship between all of the DDFS scores and frequency of the occurrence of TUTs on a daily basis and between the DDFS score and the level of controllability of the TUTs were, respectively as follows:


$$Thought-control_{ij}=\gamma_{00}+\gamma_{01}*DDFS_{j}+u_{0j}+r_{i}$$

$$TUT-frequency_{ij}=\gamma_{00}+\gamma_{01}*DDFS_{j}+u_{0j}+r_{ij}$$

### 4.2 Results

The results of the multilevel analyses are shown in **Table 5**. The models including the General Score showed that it was significantly positively related to the daily intensity of TUTs and negatively related to momentary thought control. Specific Factor 1 (Frequency) was negatively associated with thought control, but no link with TUT intensity was uncovered. Specific Factor 2 (Task-Disruptive Daydreaming) was more strongly related to both outcomes than the General Score, and its strong negative association with thought control is consistent with its content. Specific Factor 3 was not significantly associated with daily TUT intensity or thought control.

**Table 5:** Testing the link between DDFS scores and intensity of TUTs and the degree of thought control.

Factor	Task-unrelated thoughts			Thought control		
	*Coeff.*	*SE*	*t-ratio*	*Coeff.*	*SE*	*t-ratio*
General Score	0.23	0.10	2.47*	-0.47	0.12	-4.28***
Specific Factor 1	0.46	0.28	1.67	-0.97	0.34	-2.82***
Specific Factor 2	0.51	0.17	3.04**	-1.19	0.2	-5.95***
Specific Factor 3	0.36	0.32	1.14	-0.4	0.4	-1

*p <.05, ***p <.001

### 4.3 Discussion

The aim of Study 3 was to validate the DDFS using EMA. The results indicate that, in the Polish adaptation, both the General DDFS score and the Disruptive Daydreaming Factor translate well into daily intensity of TUTs and daily perceived thought control. Importantly, these findings further support the need for the three-factor solution. Factor 2, which captures the task-disruptive nature of daydreaming, shows by far the strongest associations with lower daily thought control and higher daily TUT intensity. In contrast, Factor 3 - reflecting intentional and active daydreaming, and thus less task-disruptive – did not show any associations with either thought control or TUT intensity.

## 5 General discussion

The goal of the three studies reported herein was to examine the psychometric properties of the Polish version of the DDFS (23, 24) – a popular measure used to assess the frequency of daydreaming – as well as to assess whether the use of this trait-like measure is related to participants’ everyday life experience of TUTs. There are three main findings arising from our work. First is that the Polish version of DDFS has high internal consistency and good criterion validity. Second is that the Polish version of DDFS has a bi-factor structure (with a General Factor and 3 independent Specific Factors: Frequency, Task-Disruptive Daydreaming, Non-Task-Disruptive Daydreaming). Thirdly, the DDFS score is positively related to the intensity of TUTs occurring on a daily basis and negatively related to the level of control one has over their everyday thoughts. Based on these findings, we believe that the Polish version of DDFS can be effectively used as a measure of daydreaming for research purposes in the Polish-speaking population.

Although daydreaming has been defined in multiple ways, these perspectives converge on the idea that it reflects a shift of attention away from the external task context. Because the DDFS was developed to capture this broad tendency, it remains applicable across conceptual approaches, though different definitions highlight that daydreaming may comprise partially distinct processes. Our findings support this view: the multifactor structure and differential associations with daily outcomes suggest that specific facets of daydreaming may relate to behavior in different ways. Thus, conceptual nuance should inform how DDFS scores – particularly factor scores – are interpreted in future work.

Correlations observed in Study 1 showed that daydreaming frequency was linked to depression and anxiety symptoms, consistent with existing literature (2, 9, 13). At the same time, the DDFS correlated with other measures of task-unrelated thinking – such as rumination, perseverative thinking, and future-oriented self-thought – indicating conceptual proximity among these forms of internally directed cognition. Together with the fact that, different types of daydreaming, reflected in Specific Factor 2 (Task-Disruptive Daydreaming) and Specific Factor 3 (Non-Task-Disruptive Daydreaming), show distinct associations with both psychopathology and everyday thought control, we believe that our results align with Seli’s proposal (25) that phenomena within the broader TUT family share overlapping characteristics, rather than being fully independent constructs, and are therefore best understood within a family-resemblance framework.

### 5.1 Internal consistency and criterion validity

The internal consistency of the Polish version of DDFS was found to be high, very similar to the original scale results (24), and either very similar or better than the results presented in the validation studies of other language versions (9, 34, 36). The analysis of correlation with other measures of TUTs indicates a satisfactory level of criterion validity. Additionally, the DDFS’s criterion validity was, for the first time, tested with ecological momentary assessment. This method allowed us to analyze the link between the trait-level DDFS score and the actual everyday occurrence of TUTs and control over them. The obtained results enabled us to further supplement the validation of the DDFS with additional, ecologically valid data.

### 5.2 Factor structure

As a result of the factor-structure analysis, a bi-factor solution – comprising one General Score and three Specific Factors (Frequency, Task-Disruptive Daydreaming, Non-Task-Disruptive Daydreaming) – was adopted. This structure preserves the ability to compare scores from the Polish DDFS with the original scale, while at the same time expanding the measurement to capture more nuanced characteristics of daydreaming. The distinction between Task-Disruptive and Non-Task-Disruptive Daydreaming provides information about the extent to which daydreaming interferes with ongoing activity and may therefore reflect mechanisms of executive control and contextual regulation.

### 5.3 Limitations and future directions

In Study 3 we used EMA to test the link between DFSS and everyday intensity of TUTs. Although the two are strongly related, we believe that it could be beneficial if state-level measurements were used more frequently in future research on the effects and causes of daydreaming. For that purpose, a specific validation of state-level measurements could prove useful. In the EMA protocol, the two VAS items assessed TUT intensity (i.e., the extent to which one’s thoughts diverged from the ongoing task) and subjective control over one’s thoughts. This reduced operationalization was chosen to ensure that the assessments remained brief and manageable, given the large number of measurement prompts, and to increase the likelihood that participants would accurately report their momentary thoughts. Testing of the trait level daydreaming frequency and momentary daydreaming or TUT frequency, rather than intensity, may have occurred to be more useful in this validation.

Although the factor structure identified in our data has not been reported in previous studies on the original or other adaptation of the DDFS, it appears broadly consistent with the findings of Shimoni and Axelrod (71), who showed that task-disruptiveness may be an important dimension along which laypeople conceptually distinguish between mind-wandering and daydreaming. This convergence suggests that it would be worthwhile for future research to examine whether a similar structure could also be observed in other versions of the scale.

Finally further studies need to analyze the concurrent validity of Polish scales assessing different forms of daydreaming, as the Maladaptive Daydreaming Scale (14) and the Mind Wandering Questionnaire (38) were validated after the end of the data collection for the present study. Additionally – as the sample in our study was predominantly female and non-clinical, future studies with gender-balanced samples, as well as the inclusion of a clinical sample would constitute an important complement.

### 5.4 Conclusions

Based on the results of the three studies, we conclude that the Polish version of DDFS is valid to use in the research context. Furthermore, we believe the additional EMA-based criterion applied here for validity testing represents a valuable contribution of this article to the literature concerning daydreaming and its testing, especially by considering the state aspect of daydreaming and its daily dynamics.

## Data Availability

The datasets presented in this study can be found in online repositories. The names of the repository/repositories and accession number(s) can be found below: OSF, accession cdvnf https://osf.io/cdvnf/.
