# Supplementary material for: Assessing daydreaming frequency and control with the Polish version of the Daydreaming Frequency Scale – validation using ecological momentary assessment
Source: Front Psychiatry. 2026 Feb 4;16:1694756. doi: 10.3389/fpsyt.2025.1694756 (PMC12914265; doi:10.3389/fpsyt.2025.1694756)
Supplement: Supplementary file 1 [file DataSheet1.pdf]

Supplementary Material

1. The Polish version of the Daydreaming Frequency Scale (DDFS).

1. Jak często zdarza Ci się oddawać się marzeniom?

a) Rzadko.

b) Raz w tygodniu.

c) Raz dziennie.

d) Kilka razy dziennie.

e) Wielokrotnie w ciągu dnia.

2. Marzenia dzienne i fantazje zajmują w ciągu dnia...

a) 0% moich myśli.

b) mniej niż 10% moich myśli.

c) około 10% moich myśli.

d) około 25% moich myśli.

e) więcej niż 50% moich myśli.

3. Moje myśli odpływają od tego, co aktualnie robię do innych tematów...

a) nigdy.

b) rzadko.

c) czasami.

d) często.

e) ciągle.

4. Przywołuję moje marzenia i rozmyślam o nich...

a) rzadko.

b) raz w tygodniu.

c) raz dziennie.

d) kilka razy dziennie.

e) wielokrotnie w ciągu dnia.

5. Jak często, gdy nie jesteś uważna/uważny podczas pracy, czytania książki, oglądania telewizji, Twoje myśli odpływają w świat marzeń?

a) 0% czasu.

b) 10% czasu.

c) 25% czasu.

d) 50% czasu.

e) 75% czasu.

6. Zamiast zauważać zdarzenia i ludzi wokół mnie, spędzam zagubiona/zagubiony w moich myślach około...

a) 0% czasu.

b) mniej niż 10% czasu.

c) co najmniej 10% czasu.

d) co najmniej 25% czasu.

e) co najmniej 50% czasu.

7. Jak często oddajesz się marzeniom w pracy/szkole?

a) Rzadko.

b) Raz w tygodniu.

c) Raz dziennie.

d) kilka razy dziennie.

e) wielokrotnie w ciągu dnia.

8. Przywoływanie wydarzeń z przeszłości, rozmyślanie o przyszłości lub wyobrażanie niezwykłych sytuacji zajmuje mi w ciągu dnia...

a) 0% moich myśli.

b) mniej niż 10% moich myśli.

c) około 10% moich myśli.

d) około 25% moich myśli.

e) więcej niż 50% moich myśli.

9. Intencjonalnie zatracam się w marzeniu...

a) rzadko.

b) raz w tygodniu.

c) raz dziennie.

d) kilka razy dziennie.

e) wielokrotnie w ciągu dnia.

10. Ilekroć mam na to czas, to marzę.

a) Nigdy.

b) Rzadko.

c) Czasami.

d) Często.

e) Zawsze.

11. Jak często, gdy jesteś na niezbyt interesującym spotkaniu lub widowisku, raczej nie zwracasz na nie uwagi i oddajesz się marzeniom?

a) Nigdy.

b) Rzadko.

c) Czasami.

d) Często.

e) Zawsze.

12. Jak często, gdy jesteś w długiej podróży autobusem, pociągiem, samolotem oddajesz się marzeniom?

a) Nigdy.

b) Rzadko.

c) Czasami.

d) Często.

e) Zawsze.

2. The results of item reliability tests.

Table 1S

Item reliability test for items included in the General Score Factor

Factor's Cronbach's α = .92	Item-Rest Correlation	If Item Dropped α
DDFS1	0.73	0.91
DDFS2	0.81	0.90
DDFS3	0.60	0.91
DDFS4	0.75	0.91
DDFS5	0.69	0.91

DDFS6	0.53	0.92
DDFS7	0.76	0.91
DDFS8	0.61	0.91
DDFS9	0.63	0.91
DDFS10	0.63	0.91
DDFS11	0.65	0.91
DDFS12	0.59	0.91

Table 2S

Item reliability test for items included in Factor 1

Factor’s Cronbach’s α = .9	Item-Rest Correlation	If Item Dropped α
DDFS1	0.81	0.84
DDFS2	0.76	0.87
DDFS4	0.81	0.84
DDFS7	0.71	0.89

Table 3S

Item reliability test for items included in Factor 2

Factor’s Cronbach’s α = .83	Item-Rest Correlation	If Item Dropped α
DDFS3	0.67	0.79
DDFS5	0.64	0.79
DDFS6	0.64	0.79
DDFS8	0.67	0.78
DDFS11	0.53	0.82

Table 4S

Item reliability test for items included in Factor 3

Factor’s Cronbach’s α = .77	Item-Rest Correlation	If Item Dropped α
DDFS9	0.63	0.65
DDFS10	0.67	0.61
DDFS12	0.51	0.77
