# Supplementary material for: Assessing daydreaming frequency and control with the Polish version of the Daydreaming Frequency Scale – validation using ecological momentary assessment
Source: Front Psychiatry. 2026 Feb 4;16:1694756. doi: 10.3389/fpsyt.2025.1694756 (PMC12914265; doi:10.3389/fpsyt.2025.1694756)
Supplement: Supplementary file 2 [file DataSheet2.pdf]

Supplementary Material 2

The content of the original version of the Daydreaming Frequency Scale (DDFS) (24).

1. I daydream

a) infrequently.

b) once a week.

c) once a day.

d) a few times during the day.

e) many different times during the day.

2. Day dreams or fantasies make up

a) no part of my waking thoughts.

b) less than 10% of my waking thoughts.

c) at least 10% of my waking thoughts.

d) at least 25% of my waking thoughts.

e) at least 50% of my waking thoughts.

3. As regards daydreaming, I would characterize myself as someone who

a) never daydreams.

b) very rarely engages in daydreaming.

c) tends towards occasional daydreaming.

d) tends towards moderate daydreaming.

e) is a habitual daydreamer.

4. I recall or think over my daydreams

a) infrequently.

b) once a week.

c) once a day.

d) a few times during the day.

e) many different times during the day.

5. When I am not paying close attention to some job, book or TV, I tend to be daydreaming

a) 0% of the time.

b) 10% of the time.

c) 25% of the time.

d) 50% of the time.

e) 75% of the time.

6. Instead of noticing people and events in the world around me, I will spend approximately

a) 0% of my time lost in thought.

b) less than 10% of my time lost in thought.

c) 10% of my time lost in thought.

d) 25% of my time lost in thought.

e) 50% of my time lost in thought.

7. I daydream at work (or school)

a) infrequently.

b) once a week.

c) once a day.

d) a few times during the day.

e) many different times during the day.

8. Recalling things from the past, thinking of the future, or imagining unusual kinds of events occupies

a) 0% of my waking day.

b) less than 10% of my waking day.

c) 10% of my waking day.

d) 25% of my waking day.

e) 50% of my waking day.

9. I lose myself in active daydreaming

a) infrequently.

b) once a week.

c) once a day.

d) a few times during the day.

e) many different times during the day.

10. Whenever I have time on my hands I day dream

a) never.

b) rarely.

c) sometimes.

d) frequently.

e) always.

11. When I am at a meeting or show that is not very interesting, I day dream rather than pay attention

a) never.

b) rarely.

c) sometimes.

d) frequently.

e) always.

12. On a long bus, train or airplane ride I daydream

a) never.

b) rarely.

c) occasionally.

d) frequently.

e) a great deal of the time.

From: Giambra LM. The influence of aging on spontaneous shifts of attention from external stimuli to the contents of consciousness. Exp Gerontology. (1993) 28:485–92. doi: 10.1016/0531-5565(93)90073-M
